# Composting as a Sustainable Approach for Managing Mercury-Contaminated Aquatic Biomass

**DOI:** 10.3390/toxics13070553

**Published:** 2025-06-29

**Authors:** María José Caraballo-Laza, Diana Marcela Ossa-Henao, Iván Urango-Cardenas, Mauricio Rosso-Pinto, Jean Remy Davée Guimarães, Roberth Paternina-Uribe, Yuber Palacios-Torres, José Marrugo-Negrete

**Affiliations:** 1 Faculty of Basic Sciences, Universidad de Córdoba, Carrera 6 No. 76-103, Montería 230002, Colombia; mcaraballolaza@correo.unicordoba.edu.co (M.J.C.-L.); dianaossah@correo.unicordoba.edu.co (D.M.O.-H.); 2 Department of Environmental Engineering, Faculty of Engineering, Universidad de Córdoba, Carrera 6 No. 76-103, Montería 230002, Colombia; mauriciorossop@correo.unicordoba.edu.co; 3 Tracer Laboratory, IBCCF, Federal University of Rio de Janeiro, Av. Carlos Chagas Filho 373, CEP, Rio de Janeiro 21941-902, RJ, Brazil; jeanrdg@biof.ufrj.br; 4 Faculty of Health Sciences, Universidad de Córdoba, Carrera 6 No. 76-103, Montería 230002, Colombia; rpaternina@correo.unicordoba.edu.co; 5 Natural Resources and Environmental Toxicology Group, Biology Program, Faculty of Natural Sciences, Technological University of Chocó, A.A. 292, Quibdó 270001, Colombia; yutorres86@gmail.com

**Keywords:** organic fertilizer, *Eleocharis interstincta*, mass balance, bioavailability and bioremediation

## Abstract

In this study, composting as an alternative approach for managing mercury-contaminated biomass in water bodies affected by gold mining in the Choco department was evaluated. A single-factor experiment with three treatments containing varying amounts of *Eleocharis interstincta* biomass sourced from mercury-contaminated sites was designed. During the composting process, physicochemical parameters were monitored such as temperature, pH, and electrical conductivity, while analyzing the behavior of mercury through mass balance assessments. Additionally, we determined the bioavailability of mercury in the final compost and characterized the physicochemical parameters of each compost sample. The mercury mass balance indicated a decrease in the total mercury content in the initial biomass over the composting period of 170 days. However, the total mercury concentration in the final compost increased due to the transformation and subsequent reduction of the original biomass. Mercury speciation analysis revealed that mercury was predominantly associated with the less bioavailable fractions (F4 and F5), suggesting its stabilization and low availability to biota. Therefore, the final compost has the potential to restore degraded soils by improving moisture retention, porosity, and soil fertility, thereby promoting plant growth. However, it does not fully meet the national and international technical standards for solid organic fertilizers or compost.

## 1. Introduction

Soil and water contamination by heavy metals, primarily resulting from industrial and mining activities, causes harmful effects on human and environmental health. Mercury (Hg), in particular, is of global concern due to its high toxicity, persistence, and ability to bioaccumulate and biomagnify in food webs [1]. Currently, there are various physical, chemical, and biological remediation technologies available to address this problem. Among them, phytoremediation stands out as a nature-based, low-cost solution with reduced environmental impact, using plants to remove heavy metals from soil, sediments, and water [2].

Aquatic plants have been globally recognized over the years for their phytoremediation potential due to their rapid growth, substantial biomass production, and extensive root systems, which not only enhance metal uptake from contaminated environments but also facilitate the translocation of these contaminants to their aerial tissues [3,4]. This can be considered a cost-effective and ingenious technique within the context of natural phytoremediation [5]. However, the accumulation of contaminants in macrophytes varies among plants, with the most relevant species including water hyacinth (*Pontederia crassipes* Mart.) [6], aquatic ferns, such as *Salvinia minima* Baker. [7], duckweed (*Lemna minor* L.) [8], and water lettuce (*Pistia stratoites* L.) [9]. Water hyacinth, duckweed, and water lettuce are the most frequently used floating plants for the remediation of heavy metal-contaminated water [10].

In Colombia, most aquatic ecosystems are affected by gold mining operations, with the department of Chocó being one of the regions most impacted by artisanal and small-scale gold mining (ASGM), which often releases Hg (approx. 1400 Mg/year) mainly in its elemental form (Hg^0^), leading to contamination of soil and adjacent aquatic systems (rivers, lakes, reservoirs) [11,12]. Consequently, abandoned gold extraction pits frequently exhibit elevated levels of mercury in their environmental compartments and support dense growths of aquatic macrophytes. These plants are useful for phytoremediation; however, they have the potential to bioaccumulate mercury in their roots and aerial tissues, which increases the risk of methylmercury (MeHg) formation during biomass decomposition [13]. Therefore, a challenge associated with this technique is the management of the contaminated biomass generated, which, due to inappropriate practices such as burning, burial, or abandonment in the open air, can release the accumulated metals again and become a secondary source of contamination [14].

Composting serves as a biological tool that facilitates the biotransformation of contaminants into less toxic substances or retains them within the organic matrix, thereby reducing their bioavailability [15,16]. Additionally, composting can yield various bioproducts, including organic and liquid fertilizers, which can be used as soil conditioners for degraded and contaminated soils. This process improves the physical, chemical, and biological properties of soil [17,18].

In this context, the objective of this study was to evaluate composting on a pilot scale as a strategy for the recovery of mercury-contaminated biomass by monitoring physicochemical variables, the evolution of mercury concentrations during the process, and its bioavailability in the final product.

## 2. Materials and Methods

### 2.1. Contaminated Biomass Samples

**Figure 1:**
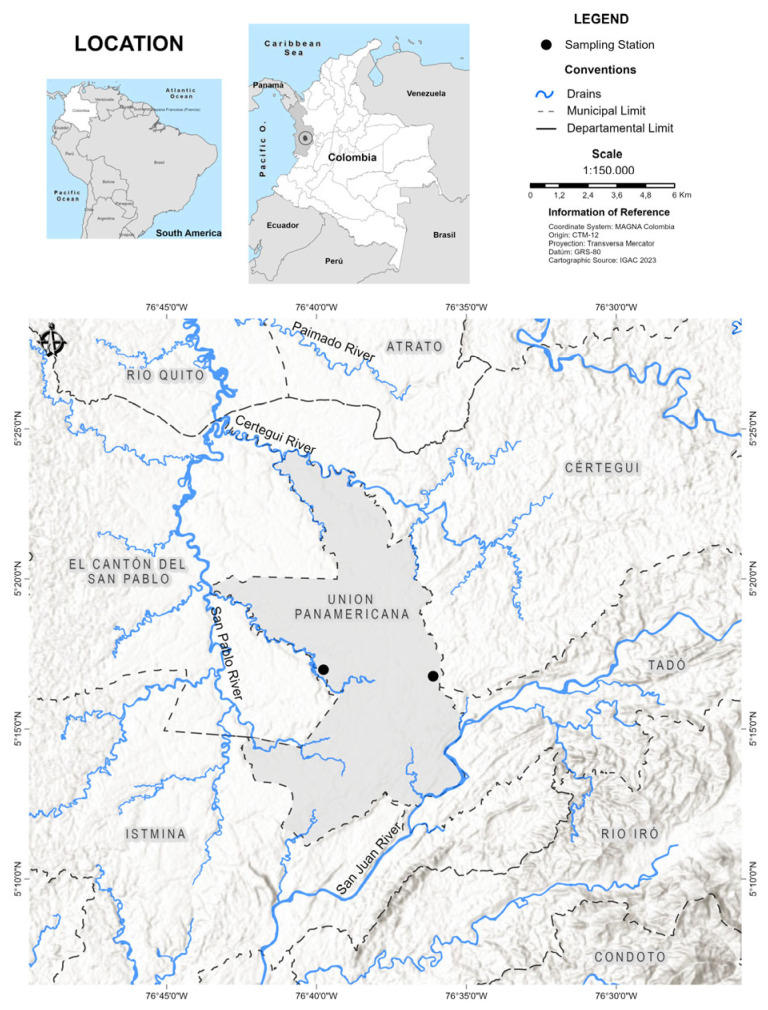
Location of the collection sites of contaminated biomass.

Mercury-contaminated biomass was obtained from two ponds abandoned after gold mining (05°16′34.9″ N; 76°35′45.9″ W and 05°17′1.7″ N; 76°39′50.7″ W) in the municipality of Union Panamericana (Choco department) in western Colombia (Figure 1). These water sources were selected based on previous studies that reported mercury (Hg) contamination [1,13]. The region’s average annual precipitation of approximately 9000 mm and an average temperature of 27 °C. Most contaminated biomass primarily consisted of specimens of *Eleocharis interstincta* (Vahl) Roem. & Schult., which was the most abundant species in the study areas.

### 2.2. Composting Process

This study employed a single-factor experimental design, with contaminated biomass as the factor and three treatments: T1 (2.5 kg), T2 (5.0 kg), and T3 (7.5 kg). A control treatment (CT) with non-contaminated biomass (NCB) was prepared for each contaminated biomass (CB) treatment to provide a reference for assessing treatment efficacy, minimizing the chance that observed differences were attributed to factors other than those in the treatments (CT1, CT2, and CT3). In total, 18 experimental units were prepared. The BC contained *E. interstincta* specimens found in two ponds in Union Panamericana, Choco. The NCB also had specimens of the same species from wetlands owned by the University of Cordoba.

The treatments varied primarily in the quantity of plant biomass incorporated into each composting unit. Additionally, supplementary inputs were added to enhance the initial compost for each treatment, including organic waste (OR, such as fruit and vegetable peels, leftover food, eggshells, and coffee grounds), livestock manure (M), and rice husk (RH), as detailed in Table 1. The pilot-scale composting process was conducted in compost bins comprising rectangular plastic boxes with a capacity of 50 kg, which were situated within a composting shed. All inputs were processed and mixed until the compost mixture was homogeneous. The initial compost mass for each treatment was 10.5 kg, including the control treatments.

### 2.3. Monitoring of Physicochemical Parameters During Composting Process

After preparing all experimental units, physicochemical parameters, such as temperature (°C), pH, and electrical conductivity (EC; dS·m^−1^), were monitored in each compost bin; enabling us to track the adequate decomposition and transformation of organic matter [19]. Monitoring was conducted twice a week over a period of five months, as was the management of the compost bins, which included extracting leachates and turning the compost to ensure proper aeration.

Temperature was measured using a bimetallic thermometer (Brixco, Milpitas, CA, USA) placed in five different locations within the compost bin to calculate the mean temperature, providing a more accurate value for each measurement. To measure the pH and EC, compost samples were collected from each compost bin (5 g). Each solid sample was placed in a 50-mL Falcon tube, and 10 mL of deionized water was subsequently added to the tube. The mixture was shaken for 1 min to dilute the sample. Subsequently, pH and EC were measured in the supernatant, adhering to the protocol outlined by [19]. Hanna pH (model pH3310/20310592) and Hanna conductivity (model Hl99300-Hl99301) (Hanna Instruments, Inc. 584 Park East Drive Woonsocket, RI 02895 United States.) meters were employed to measure the pH and EC, respectively.

#### Biomass Loss During Composting

Biomass loss resulting from the transformation of materials added in each treatment unit was quantified by calculating the quotient between the mass loss (the sum of the final mass and extracted leachates subtracted from the initial mass) and the initial mass expressed as a percentage (Equation (1)), modified from [20].

Biomass loss (%) = mi − (∑ mf + mL)/mi × 100(1)

where mi, mf, and mL represent the initial, final, and leachate masses, respectively.

### 2.4. Analysis of Total Mercury Concentrations

Total mercury (HgT) concentrations were measured at the start and end of composting in each treatment unit. The initial sample was collected on the first day of preparation of the mixture of different inputs in each treatment unit, and the final sample was collected at the end of composting. In both cases, samples were analyzed by thermal decomposition, amalgamation, and atomic absorption using a direct mercury analyzer (Milestone DMA 80 Tri Cell, Milestone S.r.l., Sorisole, Italy), according to the specifications of the Environmental Protection Agency (EPA) method 7473 [21]. Quantification was conducted using a calibration curve with a coefficient of determination of 0.9992. The limit of detection, defined as three times the standard deviation of ten blank measurements, was 0.05 ng Hg (3 μg·kg^−1^ Hg), and the method was tested using certified reference material IAEA-336, trace elements in lichens (certified value = 0.20 ± 0.04 μg·g^−1^ dry weight), and lake sediment IAEA-SL^−1^ (certified value = 0.13 ± 0.05 μg·g^−1^ dry weight) (International Atomic Energy Agency, Vienna, Austria). The recovery rates for IAEA-336 (0.19 ± 0.02 μg·g^−1^ dry weight (dw)) and IAEA-SL-^1^ (0.12 ± 0.03 μg·g^−1^ dw) were 95.0 and 92.3%, respectively, with coefficients of variation below 5%, falling within the 95% confidence intervals.

Hg content in leachates was determined using a Lumex RA-915M mercury analyzer coupled to the RP-92 module (Lumex Instruments, St. Petersburg, Russia), according to the specifications of EPA method 7470A (Hg in liquid waste by the cold vapor technique). The limit of detection of the method was 10 ng/L, and its accuracy was assessed using certified reference material SRM 1641d (mercury in water, National Institute of Standards and Technology (NIST), Gaithersburg, MD, USA), with a mean result of 1.52 ± 0.03 mg/L (*n* = 5) against a reference value of 1.557 ± 0.02 mg/L, falling within the 95% confidence intervals.

#### 2.4.1. Mass Balance for Total Mercury

Mass balance for HgT in each treatment unit was calculated using HgT concentrations at the start and end of composting, the initial and final masses of the composting mixtures, and volume and Hg concentrations in the leachates extracted in each treatment. The mass balance equation is as follows (Equation (2)):

([Hgi] × mi) = ([Hgf] × mf) + ([HgL] × VL) + mHg(2)

where [Hgi] is the initial Hg concentration (μg·kg^−1^ Hg); mi is the initial mass of the composting mixture (kg); [Hgf] is the final Hg concentration of mature compost (μg·kg^−1^ Hg); mf is the final mass of the composting mixture (kg); [HgL] is the Hg concentration in the leachates extracted in each treatment (μg L^−1^ Hg); VL is the volume of the leachates extracted in each treatment (L); and mHg is the mass of Hg lost during composting. In all cases, we reported mean values of three replicates for each treatment.

#### 2.4.2. Sequential Extraction of Mercury in the Compost

The sequential extraction method of [22] was used to determine the distribution of Hg in the different chemical phases of the final product from each treatment. This method is specifically useful for studying the mobility and bioavailability of Hg and comprises a series of five extraction fractions, wherein different forms of Hg are dissolved or selectively extracted. Hg fractionation in this method includes a water-soluble fraction (F1 or F-w), a human stomach acid-soluble fraction (F2 or F-h), organic or organo-chelated complexes (F3 or F-o), elemental Hg (F4 or F-e), and low mobility compounds, such as mercury sulfide (HgS) or mercury selenide (HgSe) (F5 or F-s). The bioavailable and most mobile fraction is derived from the sum of fractions F-w and F-h [23]. For more information, see the Supplementary Materials.

Accuracy and precision of the sequential extraction procedure were assessed by conducting the entire sequential extraction process with collected samples and reference material IAEA-SL^−1^ and subsequently comparing the sum of the phases with the total concentrations. The reference material yielded a recovery rate of 96.2 to 97.1%, and a coefficient of variation below 5% with respect to the HgT concentration, indicating high reliability of the method. HgT concentrations and other compost fractions were expressed as Hg in terms of dry weight.

### 2.5. Analysis of Compost Quality

The quality of the compost was determined according to Colombian Technical Standard 5167 (NTC-5167) for solid organic fertilizers or manures [24]. In particular, samples weighing approximately 500 g of compost were collected at the end of each composting cycle and sent to a certified laboratory for further analysis. The physicochemical parameters analyzed in the compost included pH (saturation paste/potentiometry), EC (saturation paste/conductometry), moisture retention (saturation paste/gravimetry), moisture content (gravimetry), ash content (gravimetry), total oxidizable organic carbon (TOOC; K_2_Cr_2_O solution/colorimetry), cation exchange capacity (CEC; ammonium acetate/volumetric), C:N ratio (mathematical ratio), total nitrogen (sum of nitrogen species), total phosphorus (nitric acid/colorimetric), and total potassium (nitric acid/essential amino acids (EAA).

### 2.6. Statistical Analysis

Statistical analyses were conducted to examine the behavior of the physicochemical parameters (temperature, pH, and EC) and Hg during composting. The physicochemical parameters were first analyzed using analysis of variance (ANOVA) to compare the variances between the means of different treatments. Subsequently, Tukey’s test was applied to compare all possible combinations of means and identify significant differences between them, with a significance level of *p* = 0.05. The same analyses were also applied to determine statistical differences between treatments based on the characterization parameters of the compost.

## 3. Results and Discussion

### 3.1. Behavior of the Physicochemical Parameters During Composting

#### 3.1.1. Temperature

**Figure 2:**
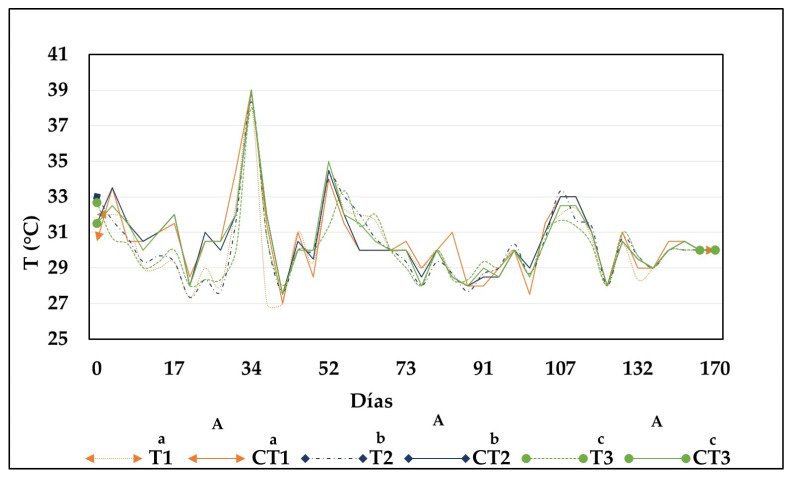
Behavior of temperature during composting in each treatment. CB treatments (T1: 25%, T2: 50%, and T3: 75%) and CT1: control treatment 1; CT2: control treatment 2; and CT3: control treatment 3. Note: Lowercase letters indicate statistical differences between each treatment with CB (T1, T2, and T3) and its respective control (CT1, CT2, and CT3). Uppercase letters indicate statistically significant differences among the different treatments (T1, T2, and T3). Different letters denote statistically significant differences (*p* < 0.05); identical letters indicate no significant differences (*p* > 0.05).

Figure 2 illustrates the behavior of temperature during composting for each treatment. Throughout the process, the behavior of this parameter was remarkably similar across treatments. However, treatments with contaminated biomass exhibited lower temperatures during the initial weeks (T1: 27.3 ± 0.6 °C; T2: 27.3 ± 0.58 °C and T3: 28 ± 0 °C) compared to control treatments (CT1: 31.5 ± 0.71 °C; CT2: 32 ± 0 °C and CT3: 32 ± 0 °C). This could be related to the adaptation of microorganisms to the presence of Hg. Conversely, the temperature in the control treatments and treatments with contaminated biomass might have been influenced by excess moisture in the compost bins, which slows down microbial activity, resulting in fewer exothermic reactions [23]. Excess moisture in the composting process of aquatic biomass is linked to the high humidity content in their structure, in addition to the contribution of water present in the fruit and vegetable residues added to the different mixtures [25,26].

The absence of a thermophilic phase in all treatments could also be related to the low volume and mass of materials in the compost bins (10.5 kg) [27,28], as well as the frequency of turning or aeration of the material, which could lead to energy dissipation (heat). Ref. [29] indicated that excessive aeration causes the compost mass to cool and become drier; therefore, an adequate turning frequency is required when working with laboratory-scale quantities. The ambient temperature could also directly affect the temperature in the compost bins as most bins exhibited similar temperature values Ref. [30] noted that temperature fluctuations during composting, especially in closed plastic compost bins, cannot be avoided owing to their low thermal inertia; thus, their internal temperature can vary throughout the day and depending on the ambient temperature.

The temperature never exceeded 39 °C during composting in all treatments, which was the maximum value measured between weeks four and five of the process. In these processes, the temperature should reach 55 °C to destroy pathogenic microorganisms and should not exceed 70 °C to avoid the inhibition of growth and metabolism of beneficial microorganisms [20,31]. At the end of composting, the temperature did not show statistically significant differences (*p* > 0.05) between treatments, with a mean temperature of 30 °C for all treatments, including their controls.

#### 3.1.2. pH

**Figure 3:**
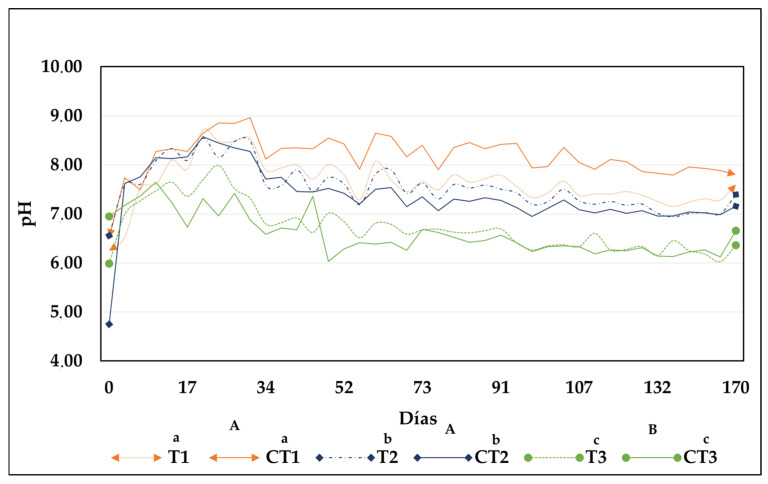
Behavior of pH during composting in each treatment. CB treatments (T1: 25%, T2: 50%, and T3: 75%) and CT1: control treatment 1; CT2: control treatment 2; and CT3: control treatment 3. Note: Lowercase letters indicate statistical differences between each treatment with CB (T1, T2, and T3) and its respective control (CT1, CT2, and CT3). Uppercase letters indicate statistically significant differences among the different treatments (T1, T2, and T3). Different letters denote statistically significant differences (*p* < 0.05); identical letters indicate no significant differences (*p* > 0.05).

During the first weeks of composting, the pH values in the treatments and their controls were slightly acidic (Figure 3), which is typical at the start of composting processes. This is due to the decomposition of soluble compounds, such as sugars, organic acids, carbon dioxide (CO_2_), and nitrates through nitrification, thereby lowering the pH [25]. As the organic acids were degraded by microorganisms to simpler compounds—carbon dioxide (CO_2_) and water—there was an increase in the pH (T1: 8.48 ± 0.28; CT1: 8.85 ± 0.01; T2: 8.48 ± 0.18; CT2: 8.35 ± 0.07; T3: 7.51 ± 0.02; CT3: 7.42 ± 0.04). The maximum pH peak coincided with the increase in temperature. Under these pH conditions, more complex carbon sources, such as cellulose and lignin, are degraded as microorganisms act by transforming nitrogen into ammonia, causing substantial increase in pH of the medium [32]. At the end of composting, the pH in treatments T1 and T2 and their controls were close to neutral (T1: 7.60 ± 0.24; CT1: 7.80 ± 0.02; T2: 7.40 ± 0.08 and CT2: 7.16 ± 0.06).

Similar results were reported by [16], who assessed the composting of Hg-contaminated organic waste and observed that the final compost, owing to its neutral pH, could be optimal for plant growth. For treatment T3 and its control, the pH remained slightly acidic at the end of the process (T3: 6.36 ± 0.09 and CT3: 6.66 ± 0.00), a behavior that could be attributed to the development of anaerobic conditions during these treatments. As a result, organic acids formed by the fermentation of organic matter may have accumulated instead of decomposing or decomposed at a slow rate [33]. Therefore, the final pH of treatments T1 and T2 showed statistically significant differences from the pH of treatment T3 (*p* < 0.05). No statistically significant differences were observed between treatments and their controls (*p* > 0.05). According to the specifications for compost in the Colombian Technical Standard 5167 [24], the pH of the final compost produced in this study falls within the acceptable range of 6 to 9, indicating that it could be used as an organic fertilizer.

#### 3.1.3. Electrical Conductivity

**Figure 4:**
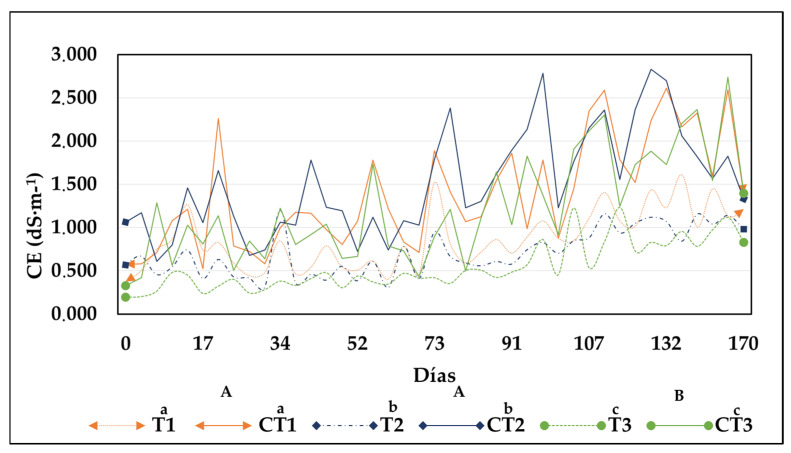
Behavior of electrical conductivity during composting in each treatment. CB treatments (T1: 25%, T2: 50%, and T3: 75%) and CT1: control treatment 1; CT2: control treatment 2; and CT3: control treatment 3. Note: Lowercase letters indicate statistical differences between each treatment with CB (T1, T2, and T3) and its respective control (CT1, CT2, and CT3). Uppercase letters indicate statistically significant differences among the different treatments (T1, T2, and T3). Different letters denote statistically significant differences (*p* < 0.05); identical letters indicate no significant differences (*p* > 0.05).

Figure 4 shows the behavior of EC in different treatments. EC indicates the content of soluble salts in the compost, and a high EC can hinder proper plant growth and seed germination [32]. On the first day, the treatments had relatively low EC values (T1: 0.376 ± 0.19 dS·m^−1^; CT1: 0.571 ± 0.04 dS·m^−1^; T2: 0.565 ± 0.54 dS·m^−1^; CT2: 1.063 ± 0.06 dS·m^−1^; T3: 0.195 ± 0.02 dS·m^−1^; and CT3: 0.326 ± 0.06 dS·m^−1^). However, EC subsequently increased considerably in control treatments, possibly owing to the nature and composition of the initial compost. EC often tends to increase during composting as the organic matter is mineralized, thereby increasing the concentration of salts [34]. In the second week of composting, EC decreased slightly, which was attributed to the leaching of secondary metabolites and waste materials generated during the mineralization of organic matter. Prior to the end of composting, EC gradually increased in all treatments. This increase could be related to the presence of ions such as Na^+^, Cl^−^, CO_3_^2−^, or SO_4_^2−^ in the composted materials [35]. At the end of composting, EC was higher in the control treatments (CT1: 1.391 ± 0.07 dS·m^−1^; CT2: 1.329 ± 0.07 dS·m^−1^ and; CT3: 1.397 ± 0.09 dS·m^−1^) than in the CB treatments (T1: 1.212 ± 0.17 dS·m^−1^; T2: 0.981 ± 0.17 dS·m^−1^ and; T3: 0.827 dS·m^−1^). The final EC values show statistically significant differences (*p* < 0.05) between the means of EC of treatments T1 (1.212 dS·m^−1^) and T3 (0.827 dS·m^−1^).

#### 3.1.4. Biomass Losses During the Composting Process

Based on Equation (1) [20], the percentage of biomass loss (%BL) was calculated for all treatments and their controls at the end of composting. Biomass losses exceeding 54% were observed in all treatments in this study, with treatments T1, CT1, T2, and CT2 showing the highest losses compared with T3 and CT3. A statistical difference was identified between the two groups of treatments (*p* < 0.05). The %BL results are shown in Table 2.

Biomass loss was greatest in treatments T1 and CT1, which could be attributed to the initial mixture of these treatments, as 60% of the biomass was comprised of organic residues, most of which were easily biodegradable materials due to their high moisture content and predominantly organic composition. Conversely, the lower biomass losses in treatments T3 and CT3 could be attributed to the complexity of the materials in its mixture as 85% of the biomass comprised plant biomass, which in turn, contained roots, stems, and leaves (75% of contaminated biomass), thereby hindering the degradation process owing to the larger quantities of roots and stems. The starting materials in a composting mixture typically comprise organic waste (such as food scraps, garden pruning waste, fruit and vegetable peels, and animal manure) that undergo rapid decomposition depending on the lignin, cellulose, or hemicellulose content in their structure, transforming into a stabilized organic matter [20]. Therefore, a common behavior in composting is the reduction in the mass and volume of organic mixtures as the process progresses, which can reach up to 70% [16]. This is mainly ascribed to the chemical transformations that occur during the biological decomposition of organic matter, which releases gases, such as CO_2_, nitric oxide and nitrous oxide (NO_x_), ammonia (NH_3_), water vapor, and other similar compounds, into the atmosphere [36,37]. Moreover, according to [38], biomass reduction during composting is also related to moisture loss in the mixtures.

### 3.2. Analysis of Hg Concentrations During Composting

Table 3 shows the behavior of the mean HgT concentrations at the start and end of composting process in treatments T1, T2, and T3. In general, the HgT concentration in the final material was higher compared with that in the initial material, indicating that Hg became more concentrated in the compost during composting process. According to [23,39,40], this behavior of Hg could be attributed to composting, as the heavy metal content increases owing to the decrease or loss of organic matter and moisture.

The mean mercury concentrations in the treatments were found to be relatively high, with values ranging from 97.96 ± 1.81 μg·kg^−1^ Hg to 112.87 ± 1.90 μg·kg^−1^ Hg at T1, and 176.44 ± 1.9 μg·kg^−1^ Hg at T3 to 216.66 ± 1.16 μg·kg^−1^ Hg at T2, and 247.56 ± 1.69 to 314.15 ± 1.54 μg·kg^−1^ Hg at T3. These values reflect the high contamination of the biomass collected from abandoned gold mining ponds in the San Juan mining district, an area particularly susceptible to elevated concentrations of Hg and other metals (Pb, Cu, Zn, Cd, Ni, Mn, and Cr) in its environmental compartments (water, sediment, vegetation, and fish) [1,13,41]. This observation underscores the substantial exposure of emergent macrophytes to mercury in these ponds. According to [1,13], the highest concentrations of mercury in macrophytes were documented in ponds with elevated levels of HgT in sediments.

HgT concentrations showed statistically significant differences between treatments (T1, T2, and T3) (*p* < 0.05). This behavior could be directly related to the initial contribution of Hg to each different treatment through the addition of different amounts of contaminated biomass, showing the following order: T3 (7.5 kg) > T2 (5 kg) > T1 (2.5 kg). This pattern is consistent with the expected increase in mercury concentration proportional to the amount of contaminated biomass used. Ref. [42] produced compost with Hg concentrations ranging from 3.16 to 6.1 mg·kg^−1^, values significantly higher than those observed in this study. These high concentrations are attributed to the grass used as the main raw material, which contained Hg concentrations ranging from 8.53 to 20.2 mg·kg^−1^. Ref. [43] also produced compost with Hg concentrations of approximately 3 mg·kg^−1^ using sludge from wastewater treatment plants with high Hg content. Although the produced compost had low Hg concentrations compared with those in other studies and complied with national and international standards for compost use with respect to Hg content, assessing the mobility rate of this contaminant is essential.

**Figure 5:**
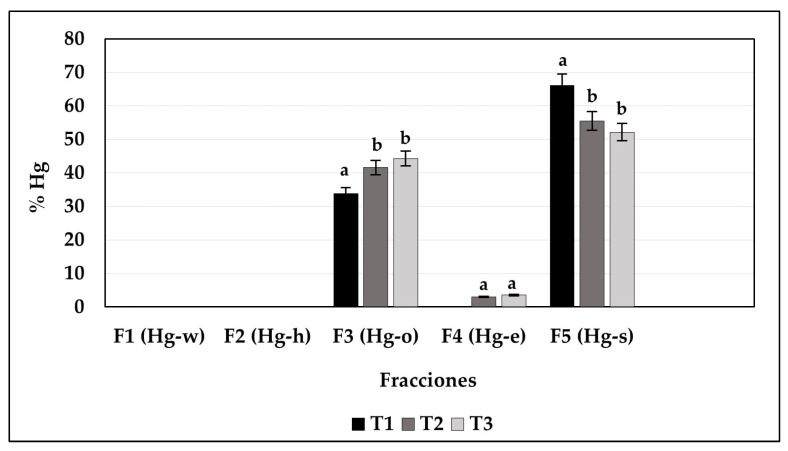
HgT fractionation in the compost of the different treatments. Notes: Letters indicate statistical differences among treatments (T1, T2, and T3) within each fraction (F1, F2, F3, F4, and F5). Different letters denote statistically significant differences (*p* < 0.05), whereas identical letters indicate no statistical differences (*p* > 0.05).

As the objective is to use this compost for agricultural soil improvement, ensuring minimal transfer of Hg through the food chain during its use is essential. This was assessed by investigating Hg fractionation according to the methodology proposed by Bloom. The results are shown in Figure 5. No bioavailable Hg was detected in the soluble fractions (F1 and F2) across all treatments. The Hg in compost produced in treatment T1 was mainly present in fractions F3 (MeHg, humic Hg, or Hg_2_Cl_2_) and F5 (HgS), representing 33.85 ± 0.25 and 66.15 ± 0.26% of HgT, respectively. The Hg in compost produced in treatments T2 and T3 was mostly present in fractions F5 or mercury sulfide (T2: 55.47 ± 0.57% and T3: 52.16 ± 0.37%) and F3 (T2: 41.58 ± 0.65% and T3: 44.22 ± 0.28%), with a small amount of Hg associated with fraction F4 or elemental mercury (Hg^0^), representing 2.95 ± 0.11% and 3.54 ± 0.16% of HgT in these treatments, respectively. Statistically significant differences were identified in the percentage of mercury associated with fractions F3 and F5 of treatment T1 compared to treatments T2 and T3 (*p* < 0.05).

The sequential extraction results indicated that Hg contained in the compost produced in the treatments was mainly associated with the residual fraction, which comprises inert and low-mobility Hg compounds (F5), in addition to being highly stable forms of mercury [22,23,44,45]. Further, a large portion of Hg found in the different treatments was associated with fraction 3 (F3), comprising Hg compounds associated with stabilized organic matter. This is expected, as compost primarily consists of stabilized organic matter. This stable organic matter favors the specific and non-specific adsorption of Hg by strongly binding to it, thereby reducing its mobility. According to [46], although it can be higher, the mobility of Hg associated with phase 3 requires extreme conditions in the environment, including the destruction of stabilized organic matter, which rarely occurs under normal temperature conditions.

Mercury stabilization during composting is a complex process influenced by chemical and microbiological interactions and the characteristics of the starting materials. Several studies have shown that, during composting, Hg tends to redistribute towards more stable chemical fractions, such as the residual and oxidizable fraction, which significantly reduces its bioavailability. According to [47], changes in pH and microbial activity during the composting process favor this immobilization, since these conditions transform the exchangeable forms of Hg into more stable and less mobile chemical fractions. This behavior has also been observed by [27] who highlight that Hg is predominantly associated with organic matter and sulfides in the oxidizable fraction, reaching up to 64.55% of the total content at the end of composting. These chemical interactions, facilitated by the humification of organic matter, are essential to reduce the mobility and environmental risk of Hg. In addition, the role of microorganisms is crucial in the transformation of mercury. Ref. [43] indicated that certain bacteria are capable of reducing Hg^2+^ to Hg^0^, thus decreasing its bioavailability and mobility.

Finally, Hg contained in the compost produced in treatment T1 was more chemically stable, as most Hg was associated with the residual fraction (F5), comprising Hg compounds that are inert and do not represent a direct risk to the environment (Figure 5). Possibly, the high content of easily degraded organic residues (60%) in the mixture provided a significant source of organic carbon, promoting the formation of humic substances that immobilize Hg [27]. This result can also be related to the findings of [48], who emphasized that the selection of biodegradable materials with high chemical interaction capacity is key to minimize heavy metal leaching and optimize compost stability. Although all treatments succeeded in concentrating Hg in the final compost, T3 showed greater efficiency, as a larger quantity of contaminated biomass was utilized and transformed during composting, while also stabilizing the Hg contained in the initial material. Therefore, composting could be a promising alternative for treating Hg-contaminated aquatic biomass as it stabilizes the metal and reduces the risk of recontamination through natural decomposition of these plants in the environment.

#### Mass Balance of Total Mercury

Considering HgT mass balance, all treatments showed a decrease in HgT during composting compared with the initial mixture. This analysis was based on the mass of Hg and not on its concentration. Considering Equation (2), Table 4 presents the decreases in Hg that occurred in the treatments (decrease in Hg is expressed as a percentage). This decrease was consistent with the findings of [44] as a clear decrease in HgT was observed in their treatments, with a 25% reduction after composting. According to [43,49], such a decrease in Hg during composting occurred mostly by volatilization, as microorganisms during composting can influence the transformation of Hg compounds and their release into the environment as vapor.

The decrease in Hg observed in the final compost in this study could also have occurred through leaching, and this was evident when analyzing their Hg contents (T1: 3.87 ± 0.01 µg·L^−1^ Hg; T2: 4.63 ± 0.15 µg·L^−1^ Hg; and T3: 6.75 ± 0.01 µg·L^−1^ Hg). Although these values did not account for all HgT lost in each treatment, they indicate the manner through which Hg can be solubilized and mobilized to be subsequently eliminated from the system, mainly in treatments where leachates were produced.

Another factor that can explain the decrease in Hg during composting is related to microbial activity, as it can influence the transformation of Hg compounds and their release into the environment as vapor [43]. The microorganisms involved in the release of Hg into the environment during composting may vary depending on the specific conditions of the process. In nature, Hg is mostly volatilized through its organic form (dimethylmercury; (CH_3_)_2_Hg) or as elemental Hg (Hg^0^) [27,50]. These species are typically formed in anoxic media with high reduction potential. The formation of (CH_3_)_2_Hg is linked to methylmercury (CH_3_Hg^+^) through the methylation of inorganic Hg (Hg^2+^) coupled with the reduction of the sulfate ion (SO_4_^2−^) (achieved by sulfate-reducing bacteria) [45,51]. Subsequently, CH_3_Hg^+^ is converted to (CH_3_)_2_Hg by abiotic processes in the presence of the sulfide ion (S^2−^) [52]. Once formed, (CH_3_)_2_Hg can volatilize at a basic pH. Hg^0^ can be formed by the biotic or abiotic reduction of inorganic Hg or the demethylation of CH_3_Hg^+^ by the action of sulfate- and nitrate-reducing bacteria or methanogens [53]. Once formed, Hg^0^ can volatilize under ambient pH and temperature conditions [54].

Hg decreased in treatments T1 and T2, both of which contained a greater amount of easily degradable inputs, which require less time to be transformed and facilitate Hg mobility into the medium. This behavior was different in treatment T3, showing a lower contribution of easily biodegradable inputs; thus, considering its nature and chemical composition, delayed degradation occurred, slowing down composting and resulting in Hg leaching. Moreover, decreases in Hg were positively correlated with biomass losses in the treatments; thus, gaseous Hg (elemental and organic) could be carried away by the water vapor produced in the transformation processes of organic matter [15,43]. Consistent with this result, the greatest decreases in Hg were mostly observed in treatments T1 and T2, with percentages of > 68 and 60%, respectively. Based on these findings and the conditions during composting, most of the decrease in Hg likely resulted from the reduction of Hg^2+^ to Hg^0^ and its subsequent emission into the atmosphere. This was mainly observed during the first weeks of the process, due to the excess moisture in the compost bins and the increased potential reduction of the reactions.

The basic pH observed during composting in some treatments could also promote decreases in Hg through the volatilization of dimethylmercury, easily formed by the methylation of methylmercury in the presence of the sulfate ion, which is a common byproduct of organic matter decomposition. The methylation of Hg during composting can also occur through the mineralization and humification of organic matter facilitated by microorganisms [49,50]. Organic matter plays a crucial role in Hg transformation as it can act as an electron donor for Hg-methylating bacteria and bind Hg to regulate its bioavailability according to the type of complexes formed. Although the organic matter content alone cannot account for all the variation in Hg methylation, its composition is essential to this process [51,53].

### 3.3. Characterization and Composition of Compost

Table 5 presents the physicochemical parameters of the compost produced in each treatment. The moisture content was above the maximum value provided by NTC-5167 in all treatments, indicating excess moisture in the compost. The moisture retention in treatments T1, T3, and controls was within the permissible range of NTC-5167, whereas that in treatment T2 was slightly below the permissible limit (98.8%). This parameter has an inverse relationship with particle size; the smaller the particles, the higher is the moisture retention capacity [55].

The pH values in all treatments were within the permissible range outlined in NTC-5167 (between 4 and 9). The slightly acidic pH observed in T3 (6.02) was presumably related to the presence of organic acids formed during composting, which accumulated instead of degrading [56]. The compost of all treatments appears suitable for agriculture use, without adversely affecting plant growth, enabling mineral solubility and nutrient availability [57].

The ash content completely complied with the specifications of NTC-5167 (<60%) in all treatments, ranging from 12.19 to 27.7%. Ash content indicates the presence of minerals in the compost and should be below 5% of dry matter. Minerals and water are components that cannot be oxidized to obtain energy; therefore, the recommended ranges of minerals and water of NTC-5167 should not be exceeded [58]. The CEC values from 35.5 to 54.1 meq·100 g^−1^ in all treatments exceeded the recommended CEC range of NTC-5167. CEC increased during composting, possibly owing to the humification that occurred during the decomposition of organic matter in the substrates [59]. The TOOC values of all treatments were above the recommended TOOC range of NTC-5167, probably owing to the low initial C:N ratio in all treatments, causing organic carbon to be transformed and released into the atmosphere as CO_2_ by microbial action [60]. The C:N ratios were within the recommended range of NTC-5167 in all treatments. The C:N ratios of treatment T1 and its control indicated lower mineralization of organic matter during composting. This could be associated with a high initial C:N ratio and the elevated carbon content, which retards the decomposition process [57].

Considering the macronutrient content, the total potassium (TK) levels complied with NTC-5167 in treatments T1 and T2 and their controls, with values ranging from 1.074 to 1.580%, whereas that in treatment T3 was below 1% (0.582%). The total phosphorus (TP) content in compost was linked to the organic matter content, specifically to organic residues [59]. Total silica levels also fell within the recommended silica range of NTC-5167 (<50%). Based on the starting material of the treatments, the maximum silica contribution is attributed to rice husks.

While some physical and chemical parameters of compost did not meet the criteria for classification of organic fertilizers, they could still be used as soil conditioners for degraded soil. While these materials do not provide a source of primary nutrients as rich as those found in commercial fertilizers, they remain beneficial for soil improvement. They offer a range of advantages in the recovery of the physical and chemical properties of soil, including moisture retention, porosity, increased organic matter content and soil fertility, microbial activity, and increased soil biodiversity, among other benefits.

## 4. Conclusions

The thermophilic phase was not reached during composting, which can be attributed to the volume of the materials, excess moisture caused by leachates, and type of compost bin used. These conditions adversely affected composting efficiency. Considering the presence of Hg in the initial biomass, although Hg concentrations were higher in the final compost in all treatments, mass balance indicated a decrease in Hg content during composting compared to that in the initial mixture, suggesting biomass losses, which was partly linked with Hg volatilization during the process. Moreover, Hg was stabilized in the final compost, mainly in the residual fraction associated with HgS and other difficult-to-degrade species, limiting its mobility and decreasing its environmental risk. The management contaminated biomass through composting has great potential, as it not only contributes to reducing the volume of contaminated waste but also stabilizes certain contaminants, such as Hg, making them less bioavailable. Although the produced compost does not meet the primary nutrient requirements of Colombian Technical Standard 5167, it is suitable as an amendment for improving the properties of degraded soil.

## Figures and Tables

**Table 1:** Inputs and their quantities for the preparation of the experimental composting units.

Treatments	* CB (kg)	* NCB (kg)	* OR (kg)	* M (kg)	* RH (kg)	Final Weight (kg)
T1	2.5	0	6	1.5	0.5	10.5
CT1	0	2.5	6	1.5	0.5	10.5
T2	5	0	3.5	1.5	0.5	10.5
CT2	0	5	3.5	1.5	0.5	10.5
T3	7.5	0	1	1.5	0.5	10.5
CT3	0	7.5	1	1.5	0.5	10.5

Notes: * CB: contaminated biomass; NCB: non-contaminated biomass; OR: organic kitchen waste; M: manure; RH: rice husk. CT1, CT2, and CT3 are the controls for treatments T1, T2, and T3, respectively. The quantity of each input is indicated in kilograms.

**Table 2:** Percentage of biomass loss in the treatments at the end of composting.

Treatments	mi * (kg)	mf * (kg)	VL * (L)	Biomass Losses (kg)	Biomass Losses (%)
T1	10.5 ± 0.1	2.84 ± 0.05	2.46 ± 0.02	5.21 ± 0.05	72.99 ± 1.50 ^a^
CT1	10.5 ± 0.1	2.88 ± 0.01	2.42 ± 0.03	5.20 ± 0.04	72.58 ± 1.19 ^a^
T2	10.5 ± 0.1	3.40 ± 0.06	1.20 ± 0.10	5.90 ± 0.16	67.66 ± 2.04 ^a^
CT2	10.5 ± 0.1	3.50 ± 0.07	1.16 ± 0.01	5.84 ± 0.08	66.67 ± 1.69 ^a^
T3	10.5 ± 0.1	4.78 ± 0.12	0.10 ± 0.01	5.62 ± 0.12	54.47 ± 1.11 ^b^
CT3	10.5 ± 0.1	4.79 ± 0.06	0.15 ± 0.06	5.54 ± 0.01	54.40 ± 1.59 ^b^

Notes: * mi: initial mass; mf: final mass; VL: leachate volume. Letters in superscript indicate statistically significant differences between treatments with respect to each measured parameter. Different letters denote statistical differences (*p* < 0.05).

**Table 3:** HgT concentrations at the start and end of composting.

CB Treatments	Initial Concentration (µg·kg^−1^)	Final Concentration (µg·kg^−1^)
T1	97.96 ± 1.81 ^a^	112.87 ± 1.92 ^b^
T2	176.44 ± 1.90 ^c^	216.65 ± 1.16 ^d^
T3	247.55 ± 1.69 ^e^	314.15 ± 1.54 ^f^

Note: Different letters are used to indicate statistically significant differences between treatment groups and variations between initial and final concentrations.

**Table 4:** Mass balance for the different treatments.

Treatments	[Hg_i_] × m_i_ µgHg	[Hg_f_] × m_f_ µgHg	[Hg_L_] × V_L_ µgHg	m_Hg lost mass_ (%)
T1	1028.6 ± 1.06	320.1 ± 1.33	9.51 ± 0.11	68.8 ± 1.68 ^a^
T2	1852.6 ± 0.20	735.6 ± 1.10	5.56 ± 0.41	60.2 ± 0.80 ^a^
T3	2599.5 ± 1.75	1501.9 ± 1.11	0.68 ± 0.02	42.2 ± 1.11 ^b^

Notes: [Hg_i_]: initial Hg concentration; [Hg_f_]: final Hg concentration; [Hg_L_]: Hg concentration in leachates; mi: initial mass of the system before composting; mf: final mass of the system after composting; V_L_: volume of leachates; m_Hg lost_: Hg lost mass. The values are expressed in µg Hg. Superscript and different letters indicate statistically significant differences (*p* < 0.05).

**Table 5:** Compost characterization and composition.

Parameters	Treatments						
	T1	Control T1	T2	Control T2	T3	Control T3	NTC-5167
Moisture (%)	**33.7 ± 2.29 ^a^**	**30.3 ± 1.18 ^a^**	**39.4 ± 1.49 ^b^**	**41.3 ± 1.34 ^b^**	**47.2 ± 0.98 ^c^**	**44.6 ± 1.11 ^d^**	**Max. 25**
Moisture retention (%)	132 ± 1.27 ^a^	137 ± 1.19 ^b^	119.5 ± 2.45 ^c^	124 ± 1.8 ^c^	103.8 ± 1.32 ^d^	110.7 ± 1.44 ^e^	**Min. 100**
pH	7.09 ± 1.19 ^a^	7.70 ± 1.10 ^bc^	7.56 ± 1.41 ^b^	7.87 ± 1.08 ^c^	7.1 ± 1.03 ^a^	7.65 ± 2.33 ^b^	**Between 4 and 9**
Ash (%)	14.05 ± 1.67 ^a^	17.7 ± 1.19 ^b^	13.55 ± 0.08 ^a^	11.8 ± 0.76 ^c^	12.19 ± 1 ^c^	10.7 ± 0.60 ^c^	**Max. 60**
CIC (meq·100 g^−1^)	42.45 ± 0.67 ^a^	47.1 ± 0.67 ^b^	38.25 ± 0.38 ^c^	34.8 ± 0.22 ^d^	35.5 ± 0.43 ^e^	33.7 ± 0.12 ^e^	**Min. 30**
COOT (%)	**14.95 ± 1.38 ^a^**	**13.9 ± 1.7 ^a^**	**11 ± 2.03 ^ab^**	**9.91 ± 0.56 ^b^**	**10.81 ± 1 ^b^**	**11.9 ± 0.88 ^ab^**	**Min. 15**
Relación C/N	15 ± 1.76 ^a^	13 ± 0.97 ^a^	17 ± 0.36 ^b^	19 ± 0.73 ^bc^	18 ± 0.75 ^b^	21 ± 0.87 ^c^	**Max. 25**
TN (%)	**0.623 ± 0.8 ^ab^**	**0.770 ± 1.66 ^ab^**	**0.557 ± 0.01 ^a^**	**0.428 ± 0.01 ^b^**	**0.580 ± 0.83 ^ab^**	**0.700 ± 0.50 ^ab^**	**Min. 1**
TP (%)	**0.282 ± 0.7 ^abc^**	**0.384 ± 0.3 ^abc^**	**0.346 ± 0.1 ^a^**	**0.209± 0.13 ^abc^**	**0.159 ± 0.02 ^b^**	**0.181± 0.05 ^c^**	**Min. 1**
TK (%)	1.074 ± 0.69 ^a^	1.580 ± 0.3 ^a^	1.145 ± 0.06 ^b^	**0.854 ± 0.25 ^b^**	**0.582 ± 0.48 ^b^**	**0.807 ± 0.73 ^ab^**	**Min. 1**
Total silicon (%)	10.1 ± 0.69 ^a^	18.6 ± 0.9 ^b^	8.2 ± 0.13 ^cd^	7.9 ± 0.21 ^c^	8.5 ± 0.28 ^d^	7.8 ± 0.31 ^c^	**Max. 50**
THg (µg·kg^−1^)	112.87 ± 1.92 ^a^	-	216.65 ± 1.16 ^b^	-	314.15 ± 1.54 ^c^	-	**Max. 17.000**

Notes: CIC: Cation Exchange Capacity; COOT: Organic Carbon Content; TN: Total Nitrogen; TP: Total Phosphorus; TK: Total Potassium. Superscript letters indicate statistically significant differences between treatments for that specific parameter. Different letters indicate statistical differences (*p* < 0.05). Values in bold are not within the recommended range of NTC-5167.

## Data Availability

Data is contained within the article.

## Supplementary Materials

The following supporting information can be downloaded at https://www.mdpi.com/article/10.3390/toxics13070553/s1. Table S1. Reagents and conditions used in the SEA process.
