# Supplementary material for: Composting as a Sustainable Approach for Managing Mercury-Contaminated Aquatic Biomass
Source: Toxics. 2025 Jun 29;13(7):553. doi: 10.3390/toxics13070553 (PMC12300872; doi:10.3390/toxics13070553)
Supplement: Supplementary file 1 [file toxics-13-00553-s001.zip › toxics-3604261-supplementary.pdf]

Table S1 provides a summary detailing the main reagents and conditions used in the SEA

Fraction (F)	Main Reagent	Specific Conditions	Description
**F1 (water-soluble)**	Ultrapure water	25°C, 1 h	Water-soluble Hg
**F2 (weak acid-soluble)**	HCl 0.1 M	40°C, 1 h	Hg soluble in weak acid
**F3 (organic-bound)**	KOH 1 M	60°C, 12 h	Hg bound to organic matter
**F4 (elemental)**	HNO_3/H_2O_2 (1:1)	80°C, 3 h	Elemental mercury
**F5 (sulfide/residual)**	Aqua regia	90°C, 3 h	Hg in sulfide form or residual fraction

process. The original methodology employed has also been referenced, allowing readers to access complete details if desired.

Table S1. Reagents and conditions used in the SEA process

Source:(1)

Bibliography

22. Bloom NS, Preus E, Katon J, Hiltner M. Selective extractions to assess the biogeochemically relevant fractionation of inorganic mercury in sediments and soils. Anal Chim Acta. 10 de marzo de 2003;479(2):233-48.
